# Clinical and molecular findings in nine new cases of tetrasomy 18p syndrome: FISH and array CGH characterization

**DOI:** 10.1186/s13039-019-0414-8

**Published:** 2019-02-08

**Authors:** Wafa Slimani, Hela Ben Khelifa, Sarra Dimassi, Fatma-Zohra Chioukh, Afef Jelloul, Molka Kammoun, Hanene Hannachi, Sarra Bouslah, Nesrine Jammali, Damien Sanlaville, Ali Saad, Soumaya Mougou-Zerelli

**Affiliations:** 1 Laboratory of Human cytogenetics, Molecular Genetics and Biology of Reproduction, Farhat Hached University Hospital, Sousse, Tunisia; 2 Neonatology Department, Hospital of Fattouma Bourguiba, Monastir, Tunisia; 3 Pediatric department, Farhat Hached University Hospital, Sousse, Tunisia; 4 Department of Child psychiatry, Fattouma Bourguiba Hospital, Monastir, Tunisia; 5 Centre de Biologie et Pathologie Est, CHU de Lyon HCL, Lyon, France; 6 Université de Sousse, Faculté de Médecine de Sousse, Hopital Farhat Hached, UR035P2, 4000 Sousse, Tunisia

**Keywords:** Tetrasomy 18p, Small supernumerary chromosome marker, FISH, Array CGH

## Abstract

**Background:**

Small Supernumerary Marker Chromosomes (sSMC) are rare chromosomal abnormalities, which have abnormal banding arrangement and take many shapes. Several disorders have been correlated with sSMC presence. The aim of this study is to characterize the sSMC derived from chromosome 18 by Fluorescence in situ hybridization (FISH) and Array Comparative Genomic Hybridization (aCGH).

**Results:**

Nine children with dysmorphic features have been investigated. They have these features in common: a triangular face, low-set ears, a large mouth with a thin upper lip, and a horizontal palpebral fissure. Epicanthus and strabismus were present in two patients. In addition, we have noticed microcephaly and mental and/or developmental delay with low birth weight. However, two patients had standard birth weight; one patient had hypospadias; two had skin problems; and three showed different congenital heart defects. One patient had corpus callosum hypoplasia. Systematic karyotype analysis revealed a de novo supernumerary chromosome. Array CGH showed a gain in copy number on the short arm of chromosome 18 in the nine cases. In one case, the sSMC seemed to be in mosaic. The breakpoints of the marker were identified using aCGH and FISH. Thus, the sSMC led to 18p tetrasomy with approximately 14 Mb lengths, between 364344 and 14763575 based on the human genome version 18.

**Conclusions:**

These results have been completed by FISH in order to ascertain the shape of the sSMC. Our results confirm the uniqueness and particularity of the iso18p syndrome on the phenotypic as well as on the genetic level.

## Background

Small Supernumerary Marker Chromosomes (sSMC) are rare chromosomal abnormalities, which can take different shapes and have abnormal banding pattern. This makes them hardly identifiable and ambiguously characterized using only conventional karyotype. They “are generally equal in size or smaller than a chromosome 20 of the same metaphase spread” [1]. Most of the sSMCs are derivatives of acrocentric chromosomes especially from chromosome 15 [2, 3]. It was found that the sSMC frequencies differ according to the group of the studied population [3]. In fact, they are found in nearly 0.044% of live births and 0.075% of prenatal cases. They are seven times more prevalent in patients with mental disabilities [3]. In 77% of the cases, the sSMC is de novo and in 23%, it is inherited from one of the parents [3]. sSMC carriers are highly variable, clinically speaking, which can be caused by many prerequisites. Chief among which are the different sizes and shapes of the marker, the presence or absence of euchromatic material, the degree of mosaicism, and the uniparental disomic profile (UPD). In fact, 70% of non-acrocentric sSMC do not have phenotypic consequences, while the other 30% have various clinical manifestations [1, 4]. Several syndromes have been associated with the presence of sSMC including Pallister Killian syndrome (OMIM 601803), Cat eye syndrome (OMIM 115470), isodicentric 15q and isochromosome18p (i18p) (OMIM: # 614290). The latter is a rare chromosomal abnormality that results in 18p tetrasomy and appears to be one of the most frequent isochromosomes observed in humans with a prevalence of 1/180000 in live born children [5, 6].

In this study, we reported the clinical and the molecular cytogenetic findings in nine Tunisian patients having isochromosome 18p. They had in common dysmorphic features, developmental delay and/or intellectual disability with slightly variable phenotypes.

## Materials and methods

### Patients

**Fig. 1:**
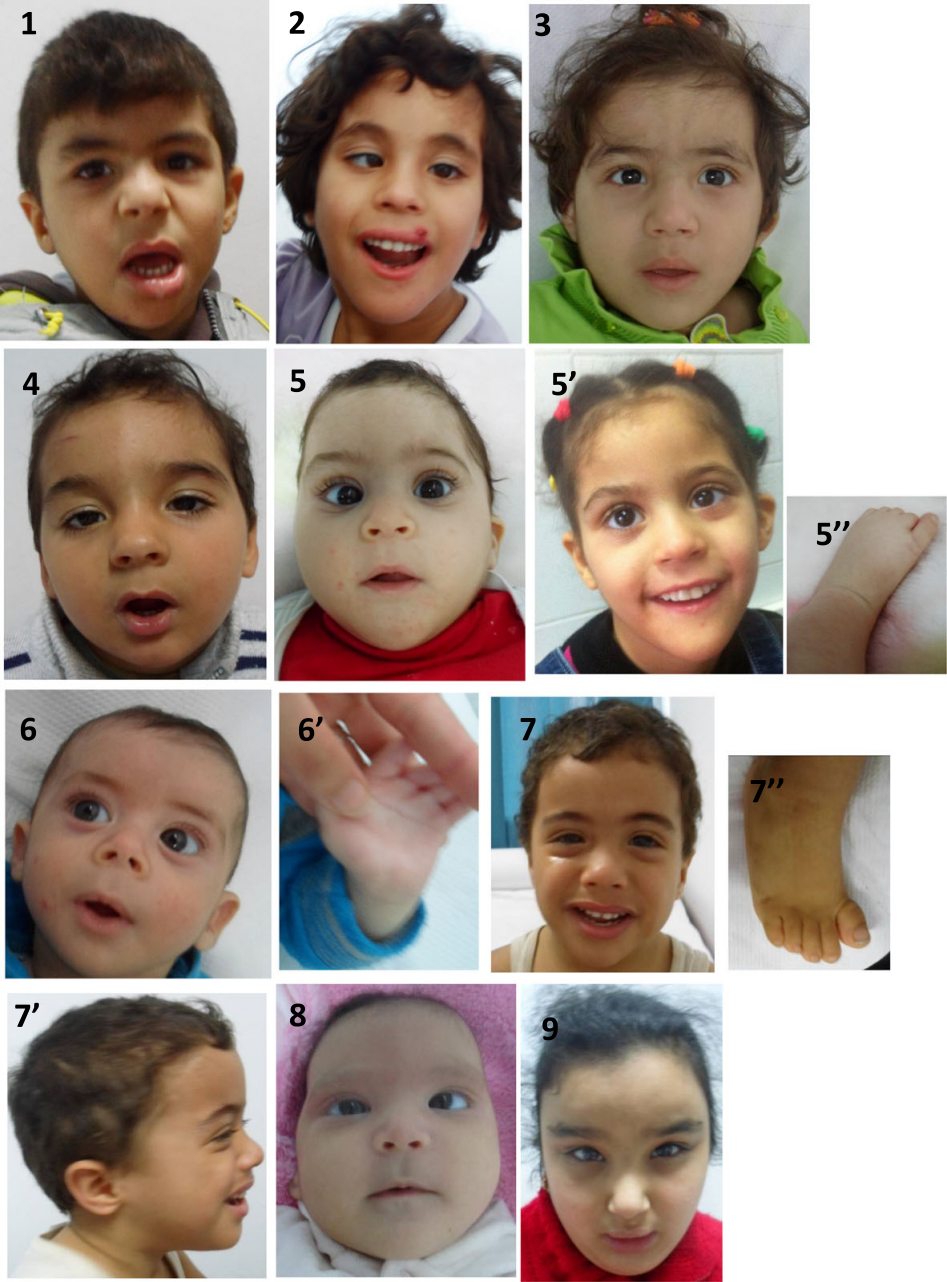
Photographs of the patients at the age of consultation (1,2,3,4,5,6,7,8 and 9), patient 5 at the age of 4 (5′), single palmar crease of patient 6 (6′), a profile picture of patient 7 (7′), Club foot seen in patient 7 (7″) and patient 5 (5″)

Nine children were referred to our department of Cytogenetics and Biology of Reproduction to explore genetic origin of their developmental delay and/or intellectual disability and dysmorphic features. All parents were also tested. The clinical findings are shown in Tables 1 and 2, and photographs are shown in Fig. 1. This study was approved by the local Ethics Board (IRB00008931) and written consents were taken from the parents for the pictures publication.

**Table 1:** Karyotypes, FISH and CGH results of the nine cases

Patient	Age (1st consultation)	Clinical manifestations	Karyotype	Final results
1	2 years	Profound ID/DF/stereotypy/hypotonia/PDA/was operated for ectopic testis	47XY+mar	47XY+mar.ish der(18)(p11.1)(wcp18+TGIF++).arr [hg18]18p11.1(364344_14378636) × 4 dn
2	6 years	GR/ID/DF/stereotypy/Strabismus/DD	47XX+mar	47XX+mar.ish der(18)(p11.1)(D18Z1+TGIF++).arr [hg18]18p11.1(364344_14584416) × 4 dn
3	2 years	GR/ID/DF/DD/MC/single palmar crease	46XX [2]/47XX+mar[18]	47,XX,+mar.ish der(18)(p11.1)(D18S552++D18Z1+).arr [hg18]18p11.1(364344_13721571) × 3~4 mat
4	2 years	GR/DF/moderate developmental delay/unexplained laughter	47XY+mar	47XY+mar.ish der(18)(p11.1)(D18S552++D18Z1+).arr [hg18]18p11.1(364344_14763575) × 4 dn
5	1 year	GR/DF/strabismus /SSL/PVS/skin problems/ seizure	47XX+mar	47XX+mar.ish der(18)(p11.1)(D18S552++D18Z1+).arr [hg18]18p11.1(364344_14763575) × 4 dn
6	5 months	GR/DF/horizontal palpebral fissures/total lack of language/axial and primary hypertension/single palmar crease/Skin problem/hypospadias/IVC	47XY+mar	47XY+mar.ish der(18)(p11.1)(D18S552++D18Z1+).arr [hg18]18p11.1(364344_14091582) × 4 dn
7	2 years	ID/FD/clubfeet/pelvicalyceal dilatation/ectopic testis	47XY+mar	47XY+mar.ish der(18)(p11.1)(D18S552++D18Z1+).arr [hg18]18p11.1(364344_14918854) × 4 dn
8	3 months	FD, DD, MC corpus callosum hypoplasia	47XY+mar	47XY+mar.ish der(18)(p11.1)(Subtel18pter++D18Z1+).arr [hg19]18p11.1(198111_14928854) × 4 dn
9	10 years	FD, Obese, ID	47XX+mar	47XY+mar.ish der(18)(p11.1)(Subtel18pter++D18Z1+).arr [hg19]18p11.1(14316_14773575) × 4 dn

Abbreviations: *ID* Intellectual Disabilities, *GR* Growth Retardation, *DD* Developmental Delay, *DF* Dysmorphic features, *MC* microcephaly, *SSL* Situs Solitus Levocardie, *PVS* Pulmonary valve stenosis, *IVC* Inter-ventricular Communication, *PDA* Patent ductus arteriosus

**Table 2:** Comparison of the clinical features of our patients with those reported by Sebold et al., 2010

Patient	1	2	3	4	5	6	7	8	9	%	Sebold et al., 2010
Clinical findings											
Sex	M	F	F	M	F	M	M	F	F		
FD											
triangular face	+	+	+	+	+	+	+	+	+	100%	*
horizontal palpebral fissure	+	+	+	+	+	+	+	+	–	89%	*
Synophris	+	–	–	–	–	–	–	–	+	22%	*
Strabismus	–	+	–	–	+	–	–	+	–	33%	45%
epicanthus	+	+	–	–	–	–	–	–	–	22%	*
low-set ears	+	+	+	+	+	+	+	+	+	100%	32%
anteverted nares	–	–	+	–	+	+	–	+	+	55%	*
Depressed nasal Bridge	+	+	+	+	+	+	+	+	+	100%	*
Smooth philtrum	+	+	+	+	+	+	+	+	+	100%	87%
small mouth	–	+	+	+	+	+	+	+	+	89%	17/31
thin upper lip	+	+	+	+	+	+	+	+	+	100%	35%
Developmental delay/ID	+	+	+	+	+	+	+	+	+	100%	100%
Growth retardation	–	+	+	+	+	+	+	–	–	78%	30%
Microcephaly	+	+	+	+	+	+	+	+	–	89%	53%
Cardiac defect	+	–	–	–	+	+	–	–	NP	37%	24%
Abnormal muscle tone	+	–	–	–	–	+	–	–	–	22%	73%
Seizures	–	–	–	–	+	–	–	–	–	11%	21%
Stereotyping	+	+	–	–	–	–	–	–	–	22%	25%
Kidney defect	–	–	–	–	–	–	+	–	–	11%	6,45%
Single palmar crease	–	+	–	–	–	+	–	–	–	22%	*
Hypospadias	–	–	–	–	–	+	–	–	–	11%	4%
Ectopic testis	+	–	–	–	–	–	+	–	–	22%	*
Hearing loss	–	–	–	–	–	–	–	–	–	0%	12%
Feet anomalies	–	–	–	–	+	–	+	–	–	22%	23%
Corpus callosum hypoplasia	*	–	–	–	–	–	–	+	*	11%	25%

+: present, −: not present, *F* female, *M* Male, *: data not collected or not mentioned, *NP* Not performed, *FD* Facial Dysmorphia, *ID* Intellectual disability

### Karyotype

The R-banded karyotypes of the patients and their parents were established at a 450-band resolution. Metaphase chromosome spreads were prepared from phytohemagglutinin (PHA)-stimulated peripheral blood lymphocytes according to standard protocol. Cell cultures were incubated for 72 h of incubation. A minimum of 15 R-banded metaphase chromosomes were analyzed for each patient using Cytovision® Karyotyping software version 4.0.

### FISH analyses

FISH was carried out on metaphase spread chromosomes using commercial probes (Vysis®, Downers Grove, Illinois, USA and Kreateck): Whole Chromosome Painting (WCP18) and *TGIF* probe, Subtelomeric (Subtelter 18p) and centromeric probe (D18Z1) for chromosome 18. TOTELVysionTM Multicolor DNA Probe Mixtures 11 (Vysis®, Downers Grove, Illinois, USA), involving the use of the same set of a different combination of probes with different colors. Probes were applied to metaphase slides. They were then co-denaturized for 7 min at 75 °C. After overnight hybridization at 37 °C and washing, chromosomes were counterstained with a 4,6 diamino-2-phenylindole (DAPI) and observed using an Axioskop Zeiss® fluorescent microscope. Images were captured with a CCD camera (Cytovision, Applied Imaging®).

### aCGH analyses

Agilent® oligonucleotide array was performed with Agilent Human Genome CGH Microarray kit 44 K and 60 K for the last two patients according to the manufacturer’s instructions (Feature Extraction 9.1, CGH Analytics 4.5, Santa Clara, California, United States). A consensus cutoff for recording an alteration was a copy number variation involving at least 3 consecutive oligonucleotides presenting an abnormal ratio greater than + 0.58 or lower than − 0.75. An in silico analysis of the unbalanced regions was made using UCSC Genome Browser (https://genome.ucsc.edu/), the Database of Chromosome Imbalance and Phenotype in Humans using Ensemble Resources (DECIPHER: https://decipher.sanger.ac.uk/), the Database of Genomic Variants (DGV: http://dgv.tcag.ca/dgv/app/home) and the Online Mendelian Inheritance in Man database (OMIM: https://omim.org/).

## Results

**Fig. 2:**
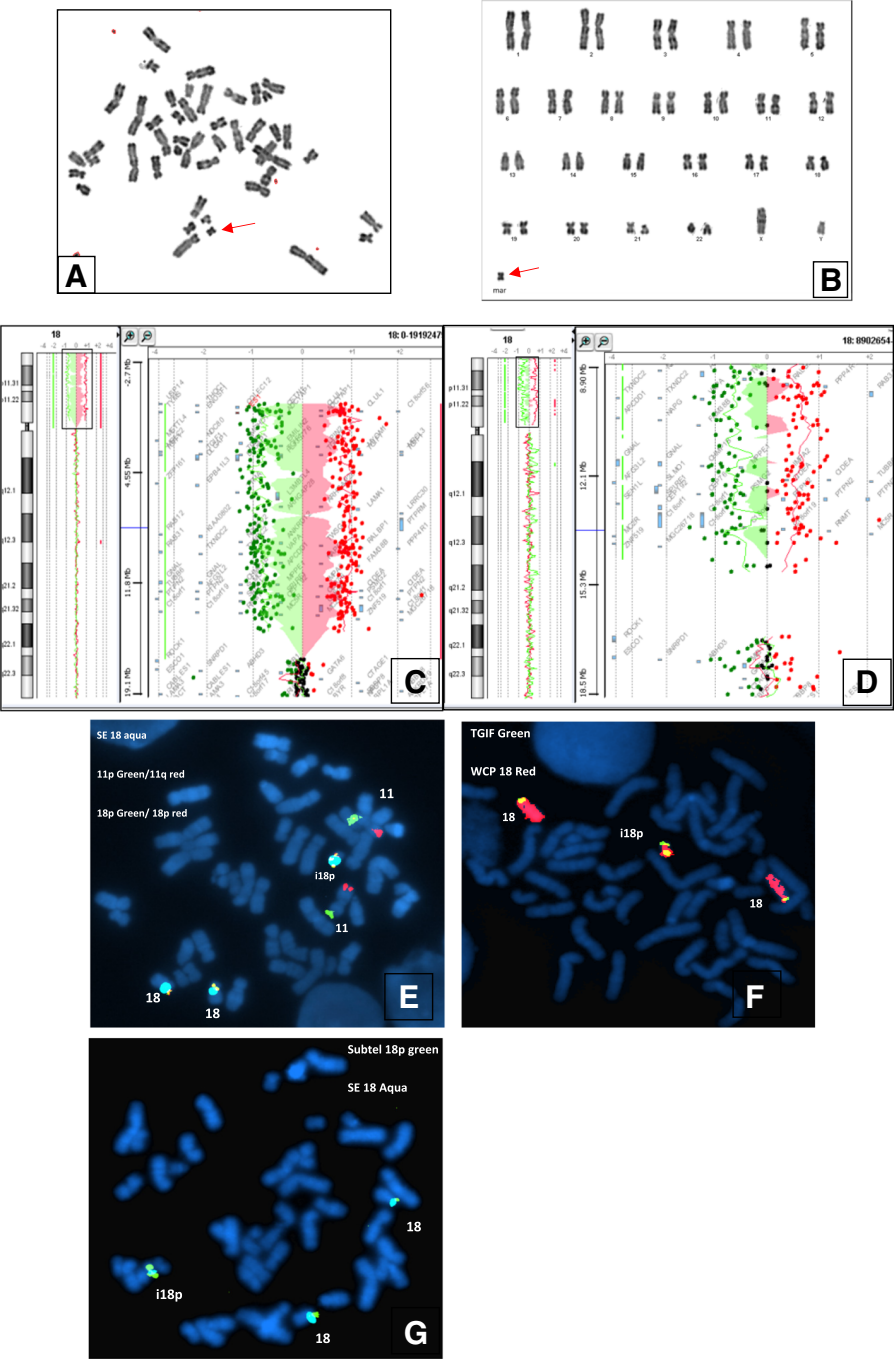
Metaphase spread (**a**) and corresponding Karyotype from patient 5 (**b**) showing a marker chromosome (Red arrow), array CGH (44 K) Results from patient 1 showing an homogenous gain of nearly the whole short arm of chromosome 18 (**c**), a mosaic duplication of nearly the same region (18p) found in patient 3 (**d**), FISH results from patient 5 using Totel Vysis Mix11 showing two normal chromosomes 18 and the isochromosome 18p (**e**), FISH results from patient 1 using *TGIF* probe (green signal) and 18p specific probe (red signal) (**f**), FISH results from patient 7 (**g**) using specific 18p subtelomeric probe (green signal) and centromere 18 (aqua)

The nine sSMCs found through routine karyotyping have been explored by aCGH using microarray with expanded coverage (44 K in seven patients and 60 K in the 2 last patients). Array CGH displayed a gain in copy numbers in 18p11 to 18pter for all the patients (Fig. 2). This result has been authenticated by a metaphase FISH analysis in order to ascertain the shape of the markers. Specific probes for chromosome 18 and ToTelVysion subtel#11 mixture probes (18p, 18q, 11p, 11q) showed an isochromosome 18p (Fig. 2). The karyotypes of all parents were normal except for patient 3. All the results are summarized in Table 1.

## Discussion

In this study, nine new cases of sSMC -originating from the short arm of chromosome 18- were reported for the first time in Tunisia. Array CGH showed a gain of nearly 14 Mb of the 18p arm with a log ratio equal to 1, resulting in its tetrasomy in eight cases. Case 3 presented a CGH log ratio equal to 0.77 indicating a possible mosaicism. In fact, FISH analyses confirmed the mosaicism in 90% of the cells with a chromosomal formula 47,XX,+mar.ish der(18)(wcp18+)[18]/46,XX [2]. The latest case is that of a 1-year-old-girl with a mild phenotype and mild intellectual disabilities. Follow-up showed a very sociable child with more discrete dysmorphic features. Accordingly, the mild phenotype was due to the level of mosaicism of the i18p. In fact, somatic mosaicism has been frequently studied and reported in patients with sSMC [7]. Actually, about 50% of the reported cases are in mosaic [7]. It seems to be related to the fact that the mosaicism level in different human tissues is very variable and unpredictable. However, this fact is rarely reported in isochromosome 18p [8]. Moreover, i18p occurs usually de novo and only a few studies reported familial cases of isochromosome 18p [7–9] which is in accordance with the present study. In fact, only one case (case 3) out of nine was in mosaic and inherited from a phenotypically healthy mother.

Previous case reports and studies of larger cohorts allowed to delineate the phenotype of tetrasomy 18. The largest series described is a multicentric study in collaboration with the Chromosome 18 Clinical Research Center [5]. In this collaborative study, Sebold et al., reported the molecular and clinical findings in 43 cases of tetrasomy 18p [5]; then five years later the same team reported the cognitive and behavioral characteristics [10]. The clinical findings of this disorder are now recognized as a distinct phenotype including moderate to severe intellectual disabilities [5, 10], growth retardation, microcephaly, strabismus, abnormalities in muscle tone, scoliosis/kyphosis, neonatal jaundice, recurrent otitis media, hearing loss, seizures, refractive errors, a history of constipation and gastro esophageal reflux, heart defects, and pes planus [5]. Other clinical features such as developmental delay and cognitive impairment can also be seen. Less frequently, kidney defects, hernias, and myelomeningocele as well as short stature and modifications are observed on Magnetic Resonance Imaging (MRI) [5]. In the present study, patients had in common dysmorphic features including a triangular face, low-set ears, a thin upper lip, and horizontal palpebral fissures. Epicanthus and strabismus were present in two patients (patient number 2 and 5). Microcephaly and intellectual disabilities and/or developmental delay with low birth weight were also present. Two patients (patient 1 and 9) presented standard birth weight, though. We also noticed that one patient had hypospadia (patient 6); two (patient 5 and 6) had skin problems; three showed a congenital heart defect (patient 1, 5 and 6) and one (patient 7) had kidney defect and ectopic testis. Brain MRI showed a hypoplastic corpus callosum in patient 8. Clinical manifestations seen in our patients are summarized and compared to those reported by Sebold et al. [5], in Table 2.

Array CGH showed the same centromeric origin in the nine SMCs corresponding to the first oligonucleotide on the DNA chip on the short arm of chromosome 18 and nearly the same breakpoints. This confirms the distinctive clinical features of isochromosome 18p syndrome (i18pS). However, we have noticed a small variation in the telomeric side, which can be either a redesign of this region or an artefact reflecting the limited specificity of the hybridization at the telomeric regions by the aCGH technique. Indeed, genes are infrequent in the 18p telomere and undoubtedly, the chip is poorly enriched with oligonucleotides in this region.

In this study, aCGH showed also five CNVs in three patients. Nevertheless, we could not associate them with any abnormality observed in our patients. In fact, based on Palmer’s criteria, those CNVs were all recorded as polymorphisms mainly with the unavailability of the parents [11].

Curiously, among the series of 39 sSMC collected since 2006 in our Laboratory (data not shown), i18p seems to be the most frequent sSMC followed by derivative sSMC 15 unlike what was reported in the literature [2, 3, 12]. This is mainly accounted for by the small size of our cohort and also by the fact that many cases of sSMC 15 could be missed when the phenotype is normal. Indeed, around 70% of cases with sSMC 15 are phenotypically normal and can go unnoticed through generations [13].

Ever since its expansion, aCGH had a huge bearing on the identification and delineation of new syndromes. It is not only much more sensitive and efficient than the conventional karyotype but it also offers a 10-time higher chromosomal anomalies diagnosis and could also, in many cases, give a genetic explanation for the learning and developmental difficulties. In case of euchromatic sSMC, aCGH can easily characterize the exact size, the origin, the breakpoints as well as the gene content in a single step; thereby, enabling a better genotype-phenotype correlation [14]. Besides, it can detect additional polymorphisms allowing a better delineation of one patient’s genotype [15]. On the other hand, aCGH has some limitations; it cannot detect balanced rearrangements, inversion, and low level of mosaicism (< 10 to 20%). Another drawback of aCGH is that the centromeric and heterochromatic regions are not covered [16]. Consequently, acrocentric and heterochromatic sSMC could be overlooked by aCGH [16]. Despite the fast progress of molecular techniques, such as Whole genome sequencing (WGS) and aCGH, and their high sensitivity, the conventional karyotype technique and FISH still are still as important as ever in the detection of sSMC and other balanced chromosomal rearrangements especially when the clinical manifestations are highly evocative of a known syndrome.

## Conclusion

This is the first study in Tunisia to report the clinical and molecular findings in nine patients having tetrasomy 18p syndrome. So far, according to Dr. Liehr’s database (Liehr T. 2018. Small supernumerary marker chromosomes. http://ssmc-tl.com/sSMC.html [accessed 15/11/2018]), 393 sSMC originating from chromosome 18 have been reported including 320 cases of isochromosome 18p. Our study expands the cohort of patients, the isochromosome 18p of whom, is molecularly characterized; and contributes to a better understanding of the genotype-phenotype correlation of tetrasomy 18p syndrome.

## Abbreviations

CGH: Comparative genomic hybridization
CNV: Copy Number Variations
DAPI: diamino-2-phenylindole
DD: Developmental Delay
DF: Dysmorphic features
DNA: deoxyribonucleic acid
FISH: Fluorescence in situ hybridization
GR: Growth Retardation
i18p: Isochromosome 18p
ID: Intellectual Disabilities
IVC: Inter-ventricular Communication
MC: microcephaly
MRI: Magnetic Resonance Imaging
PDA: Patent ductus arteriosus
PHA: phytohemagglutinin
PVS: Pulmonary valve stenosis
SSL: Situs Solitus Levocardie
sSMC: Supernumerary marker chromosomes
UPD: uniparental disomy
WCP: Whole Chromosome Painting
WGS: Whole genome sequencing
